# Developing food label literacy among adolescents

**DOI:** 10.3389/fped.2026.1741062

**Published:** 2026-04-09

**Authors:** Firuza Damirkhanli

**Affiliations:** Department of Pediatrics, Ankara Medipol University, Ankara, Türkiye

**Keywords:** adolescent health, adolescent nutrition, food label literacy, food literacy, health education, preventive pediatrics

## Introduction

Although adolescent nutrition is widely recognized as a cornerstone of long-term health, there is a striking lack of literature specifically addressing food label literacy education for adolescents in general pediatric practice. Most available research and interventions are limited to adolescents with chronic conditions such as type 1 diabetes, celiac disease, or severe allergies. However, the broader adolescent population—who are at equal risk of developing poor dietary habits—remains largely overlooked in both research and clinical education. This commentary aims to highlight this critical gap and advocate for the integration of brief, accessible food label literacy interventions into routine pediatric care, regardless of medical diagnosis.

Adolescence is a pivotal stage for establishing lifelong health behaviors, including dietary patterns. Poor dietary habits during this period—particularly high consumption of packaged and ultra-processed foods—are associated with obesity, dyslipidemia, hypertension, and increased long-term risk of cardiovascular and metabolic diseases (1, 2). Despite these risks, preventive nutrition education in routine pediatric practice remains limited, and food label literacy is rarely addressed outside disease-specific counseling.

## Gaps in preventive nutrition for adolescents

Current nutrition counseling practices in pediatric settings primarily target adolescents diagnosed with conditions such as type 1 diabetes, celiac disease, or severe allergies. This selective approach overlooks the broader adolescent population, potentially missing critical opportunities to instill foundational health literacy. Adolescents who frequently consume packaged foods high in saturated fats, added sugars, sodium, and food additives are at increased risk for obesity, insulin resistance, elevated blood pressure, and early atherosclerotic changes (1, 2). Nevertheless, food label use is not routinely encouraged during standard pediatric visits.

## A practical and scalable approach

In routine pediatric outpatient settings—particularly well-child and preventive care clinics—food label literacy education can be delivered as a brief, structured intervention targeting the adolescent population. Pediatricians are uniquely positioned to provide this education during regular growth monitoring visits, vaccination appointments, and school health assessments, where nutrition and lifestyle counseling are already expected components of care.

In clinical practice, this intervention may be implemented within approximately 2–3 min and does not require additional personnel or educational materials. Using commonly consumed packaged foods as visual examples, pediatricians can demonstrate how to read and interpret key elements of food labels, including serving size, added sugars, saturated fat, sodium content, and selected food additives. The emphasis should be placed on helping adolescents recognize products with excessive amounts of these components and understand their potential health implications.

Evidence supports the effectiveness of front-of-package labeling systems in improving consumer understanding and supporting healthier food choices (3).

The primary goal of this intervention is to enhance awareness around food choices rather than to promote restrictive eating behaviors. When delivered in an age-appropriate, balanced, and non-judgmental manner, brief food label education in pediatric settings may support informed decision-making while minimizing the risk of disordered eating patterns, including orthorexia nervosa (4).

The World Health Organization identifies adolescence as a critical developmental period for preventive health interventions, particularly in relation to noncommunicable disease risk factors (5). WHO highlights that behaviors established during adolescence—including dietary patterns—have long-term implications for adult health outcomes. Within this preventive framework, strengthening adolescents' capacity to make informed dietary decisions represents an important component of broader public health strategies.

## Addressing ethical concerns and inclusivity

It is essential that food label literacy interventions are delivered with sensitivity. Overemphasis on ingredient avoidance or rigid definitions of ‘healthy eating' may contribute to disordered eating behaviors, including orthorexia nervosa. Educational messaging should therefore focus on awareness, moderation, and informed choice, and be applicable to all adolescents regardless of body weight or socioeconomic background.

## Conclusion and call to action

Pediatricians play a central role in shaping long-term health behaviors. Integrating food label literacy into routine pediatric outpatient care represents a feasible, low-cost, and scalable preventive strategy. By moving beyond diagnosis-driven nutrition counseling and adopting proactive, inclusive education, pediatric care can better support adolescents in developing lifelong awareness related to healthier food choices.

School-based survey studies may allow for an objective assessment of adolescents' knowledge, attitudes, and awareness regarding food label literacy. Findings derived from such surveys could inform the planning and implementation of brief, age-appropriate educational programs. Studies designed in this way may contribute to evaluating the feasibility and effectiveness of these interventions, while also providing guidance for strengthening, standardizing, and disseminating such programs across different school and clinical settings. Throughout this process, it is important that educational content is delivered within a balanced and non-restrictive framework to support informed food choices without increasing the risk of disordered eating.
